# Transforming Patient Experience: Real-World Impact of Mepolizumab on Symptom Burden in Chronic Rhinosinusitis with Nasal Polyps—A Multicenter Perspective

**DOI:** 10.3390/jcm14155248

**Published:** 2025-07-24

**Authors:** Alfonso García-Piñero, Tomás Pérez-Carbonell, María-José Gómez-Gómez, Encarna Domenech-Campos, Fernando Martinez-Expósito, Noelia Muñoz-Fernández, Jordi Calvo-Gómez, Carmen García-Navalón, Lucas Fito-Martorell, Felip Ferrer-Baixauli, Ainhoa García-Lliberós, Nezly Mosquera-Lloreda, Chakib Taleb, Carlos Zac-Romero, Cecilia López-Valdivia, Juan Pardo-Albiach, Miguel Armengot-Carceller

**Affiliations:** 1 Rhinology Unit, Hospital Universitari i Politècnic La Fe, 46026 Valencia, Spain; munyoz_noe@gva.es (N.M.-F.); fito_luc@gva.es (L.F.-M.); mosquera_nez@gva.es (N.M.-L.); 2 Rhinology Unit, Hospital Clínico Universitario, 46010 Valencia, Spain; perez_tomcar@gva.es (T.P.-C.); calvo_jorgom@gva.es (J.C.-G.); ferrer_fel@gva.es (F.F.-B.); taleb_cha@gva.es (C.T.); 3 Rhinology Unit, Hospital General Universitario, 46014 Valencia, Spain; gomezmjosegomez@gmail.com (M.-J.G.-G.); carmengnavalon@hotmail.com (C.G.-N.); garcia-lliberos_ain@gva.es (A.G.-L.); 4 ENT Department, Hospital Arnau de Vilanova, 46015 Valencia, Spain; domenech_marcam@gva.es; 5 ENT Department, Hospital Francesc de Borja, 46702 Gandía, Spain; 6 Pathology Department, HUiP La Fe, 46026 Valencia, Spain; zac_car@gva.es (C.Z.-R.); lopez_cec@gva.es (C.L.-V.); 7 Department of Mathematics, Physics, and Technological Sciences, Universidad Cardenal Herrera-CEU, CEU Universities, 46115 Valencia, Spain; juaparal@uchceu.es; 8 ENT Department, University of Valencia, CIBERES, BMCG, IIS La Fe, 46010 Valencia, Spain

**Keywords:** CRSwNP, mepolizumab, smell, nasal congestion, SNOT-22, patient-reported outcomes, multicenter study, real-life

## Abstract

**Background**: Chronic rhinosinusitis with nasal polyps (CRSwNP) is a chronic upper airway disease that may involve different inflammatory endotypes, although in Western populations it is most commonly associated with type 2 inflammation. CRSwNP has a significant impact on the patient’s quality of life. The recommended appropriate medical therapy is effective in controlling CRSwNP symptoms in many patients; however, a subset continues to exhibit persistent type 2 inflammation, evidenced by recurrent nasal polyps, elevated eosinophil counts, or the need for systemic corticosteroids or surgery. Monoclonal antibodies have recently become a novel and personalized treatment that can help refractory patients restore disease control. **Objective**: The present study aims to evaluate the effectiveness of mepolizumab in real-world settings in a diverse patient population, focusing on assessing the impact of this therapy on patient-reported outcomes after six months of treatment. **Methods:** This is a multicenter, observational study of CRSwNP patients treated with mepolizumab carried out in five hospitals located in Spain. Adult patients with a diagnosis of uncontrolled CRSwNP were included in the study. The change in the nasal polyp score (NPS) was the main clinical endpoint. Changes in the Sinonasal Outcome Test (SNOT-22), nasal congestion and smell impairment visual analogue scale scores, and blood and nasal polyp tissue eosinophil counts were among other endpoints included. **Results**: In total, 47 patients were included, and 91% were asthmatic. The nasal polyp score (0–8) was reduced significantly in the cohort (mean change: −2.56, *p* < 0.0001). The mean SNOT-22 score improved 25.29 points. Nasal congestion (−3.57, *p* < 0.0001) and smell impairment (−4.0, *p* < 0.0001) visual analog scale scores (0–10) showed a significant improvement. Blood and tissue eosinophil median counts showed significant reductions versus baseline of 86% and 26%, respectively. Among those patients with asthma, the asthma control test score achieved a median value of 24 points. **Conclusions**: This study provides real-world evidence supporting the effectiveness of mepolizumab in managing CRSwNP in patients with features suggestive of type 2 inflammation. The observed improvements in patient-reported outcomes, nasal polyp burden, and asthma control suggest that mepolizumab may be a valuable therapeutic option for this patient population.

## 1. Introduction

In our clinical setting, chronic rhinosinusitis with nasal polyps (CRSwNP) is an upper respiratory disease predominantly driven by type 2 inflammatory mechanisms managed with daily intranasal corticosteroids and saline rinses [1]. The most burdensome patient-reported symptoms are nasal congestion, rhinorrhea, and smell impairment, which severely impacts patient’s quality of life (QoL) to a similar extent than other devastating chronic diseases such as COPD or asthma [2]. Most patients successfully control CRSwNP symptoms with the recommended appropriate medical therapy, whereas some fail to control inflammation and require recurrent courses of systemic corticosteroids (SCS) and endoscopic sinus surgery (ESS) [1]. However, overreliance on systemic corticosteroids comes with short- and long-term detrimental adverse effects for patients. While ESS reduces local inflammatory burden by removing nasal polyps and diseased mucosa, it does not directly address the underlying type 2 immunological mechanisms responsible for disease persistence or recurrence [3]. Monoclonal antibodies (mAb) have recently emerged as an innovative, personalized treatment that allows refractory patients to restore disease control. These biologic therapies act on blocking key molecules involved in type 2 inflammation, such as IL-4/IL-13 (dupilumab, anti-IL-4r mAb) [4], IgE (omalizumab, anti-IgE mAb) [5], and TSLP (tezepelumab, anti TSLP mAb) or IL-5 (mepolizumab [6] and long-acting depemokimab, anti-IL-5 mAb). Only dupilumab, omalizumab, and mepolizumab have been approved for RSCwNP treatment.

In the SYNAPSE study, a 52-week, placebo controlled, phase III randomized clinical trial in severe CRSwNP patients [6], mepolizumab demonstrated a reduction in endoscopic nasal polyp score (NPS), improvement in patient-reported outcomes such as chronic rhinosinusitis quality of life-related test Sinonasal Outcome Test-22 items (SNOT-22), nasal congestion and smell impairment assessed using the visual analog scale (VAS), as well as a reduction in the need of ESS and SCS use. The wealth of evidence regarding the effectiveness of these therapies in addressing the most disturbing symptoms reported by patients in clinical settings is scarce, and it is mainly focused on subjects with comorbid uncontrolled severe asthma [7,8,9,10,11,12,13].

The present study aims to evaluate the effectiveness of mepolizumab in real-world settings in a diverse patient population, focusing on assessing the impact of this therapy on patient-reported outcomes after six months of treatment. In addition to reporting conventional clinical outcomes, this study offers a detailed analysis of patient-reported symptom burden using the SNOT-22 instrument, including domain-specific and item-level evaluation, which has been rarely described in prior real-world reports [14].

## 2. Materials and Methods

### 2.1. Study Design and Population

This is a multicenter, observational study of CRSwNP patients treated with mepolizumab carried out in five hospitals located in Spain. The inclusion criteria were adult patients with a diagnosis of uncontrolled CRSwNP despite appropriate medical treatment as per EPOS 2020 guidelines and treated with mepolizumab for the indication of CRSwNP according to disease management guidelines [1,15]. According to EPOS-EUFOREA 2023 and national Spanish guidelines (e.g., POLINA 2.0), systemic corticosteroid use is one of several criteria for biologic eligibility in CRSwNP. Although 81% of our patients required SCS in the prior year, all included patients fulfilled the minimum criteria required for biologic indication, even in cases without recent SCS use. Although most patients had comorbid asthma, the indication for initiating mepolizumab was, in all cases, uncontrolled CRSwNP, as per EPOS 2020 and national Spanish POLINA guidelines [16]. All included patients demonstrated type 2 inflammation using multiple biological markers prior to treatment initiation, such as elevated blood eosinophil counts, tissue eosinophilia in nasal polyp biopsies, and presence of comorbid conditions typically associated with this endotype (e.g., asthma, allergic rhinitis, non-steroidal anti-inflammatory drug-exacerbated respiratory disease [N-ERD]).

The decision to initiate mepolizumab therapy was made on an individual basis by a multidisciplinary committee consisting of otolaryngologists, allergists, pulmonologists, and hospital pharmacists. In all included cases, the choice of mepolizumab was based primarily on the patient’s inflammatory profile, which was consistent with type 2 inflammation, and on the presence of associated type 2 comorbidities such as eosinophilic asthma, allergic rhinitis, or N-ERD. All patients exhibited elevated blood eosinophil counts and/or eosinophilic infiltration in nasal polyp tissue. Although other biologics were also available in our healthcare system, mepolizumab was considered the most appropriate therapeutic option in these specific patients due to its targeted mechanism of action and established efficacy in eosinophilic-driven disease. Subjects were excluded if they were receiving another concomitant biologic treatment for the same condition. Patients were required to have available clinical records at least 12 months before mepolizumab initiation and at least 6 months after the initiation of treatment. Patients were consecutively included in the study, and data were retrospectively collected from electronic clinical records between April and November 2024. The study was conducted in accordance with the Declaration of Helsinki, and the protocol was reviewed and approved by the ethics committee of the principal investigators’ institution (Project identification code: MEPO-SINVAL-23) on 24 April 2024 and was subsequently endorsed by the ethics committees of the collaborating centers before the start of data collection. The exemption from informed consent proposed for this study was approved by the ethics committee of our institutions in accordance with local legislation (Law 14/2007, of July 3, on Biomedical Research (Spanish Biomedical Research Act)).

Baseline values for all clinical, laboratory, and patient-reported outcome measures were defined as those recorded within the 30 days prior to the initiation of mepolizumab treatment.

### 2.2. Clinical Endpoints

The primary endpoint was the change in NPS at six months of mepolizumab therapy compared with baseline values. Each nostril was scored on a scale of 0 to 4, with the total score being the sum of left and right nostril scores (range: 0–8) as evaluated using nasal endoscopy. Secondary endpoints included the change in SNOT-22 sinonasal-related quality of life test score, nasal congestion VAS, smell impairment VAS, blood eosinophil count, and nasal polyp tissue eosinophil count, evaluated at six months vs. baseline values respectively. Smell impairment and nasal congestion severity were stratified as follows: mild (VAS ≤ 3), moderate (3 < VAS ≤ 7), and severe (VAS > 7). Prescribed courses of SCS and ESS events indicated for the control of CRSwNP were registered during the data collection period. In patients with concomitant diagnosis of asthma, the change in Asthma Control Test (ACT) score was also assessed. Mepolizumab-related adverse events as per investigator criteria were collected throughout the study. The Spanish validated version of the SNOT-22 by De Los Santos et al. was used in this study [17].

### 2.3. Statistical Analysis

Descriptive and inferential statistical analyses were performed using different libraries in RStudio software (version ‘2024.12.0.467’, RStudio, Boston, MA, USA) [18]. Qualitative variables were represented as percentages, and McNemar’s chi-squared test with Yates continuity correction was used to analyze paired nominal data. The quantitative variables were analyzed using a paired sample *t*-test. When the assumptions of applicability of this technique were not met, the Wilcoxon signed rank test with continuity correction was used instead. Additionally, to compute the 95% confidence interval of the difference obtained before and after the intervention, the Hodges–Lehmann estimator was calculated.

Finally, to describe the magnitude of the difference or relationship between the means of two paired groups, the effect size was calculated using Cohen’s D with Hedges Correction. It quantifies the standardized difference between the means of two paired groups. It is calculated by dividing the mean difference between the paired observations by the standard deviation of those differences. It indicates the practical significance, or the strength of the relationship observed, independent of the sample size (0.2 = small, 0.5 = medium, and 0.8 = large effect).

## 3. Results

### 3.1. Baseline Characteristics

A total of 47 patients with a mean age of 54 years were included in the study (Table 1). Patients had a mean age of 38.4 years when CRSwNP was diagnosed and a mean elapsed time of approximately 15 years from diagnosis to initiation of biologic treatment. ESS was performed in 83% of the subjects. Mean time from the last surgery to the initiation of mepolizumab was around 5 years. Asthma was present in 91% of the cohort, with roughly a similar proportion of subjects classified as having severe and non-severe asthma. In total, 81% required SCS prescription in the 12 months before mepolizumab treatment. The five most burdensome symptoms experienced by the studied cohort, ranked by order based on baseline SNOT-22 frequency of selection, were loss of taste/smell (11.9%), need to blow nose (11.4%), nasal congestion (10.8%), runny nose (8%) and thick nasal discharge (8%) (Figure S1).

### 3.2. Nasal Polyp Score

**Figure 1:**
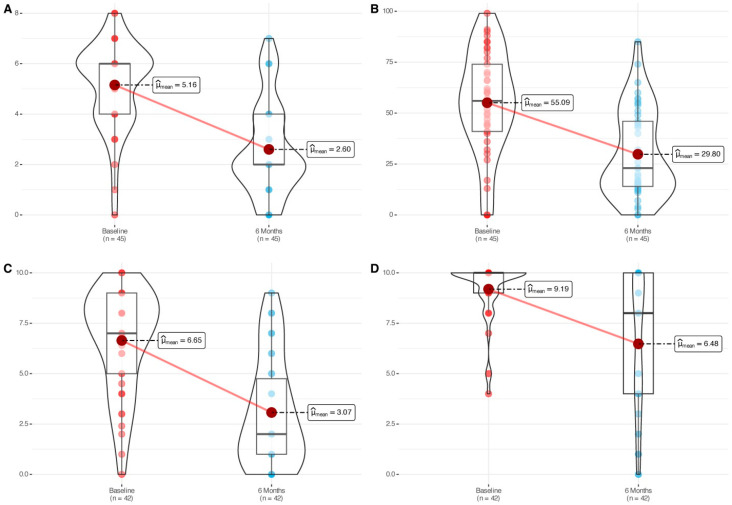
Improvements in (**A**) total NPS; (**B**) SNOT-22; (**C**) nasal congestion VAS; (**D**) smell impairment VAS at baseline and after six months of mepolizumab treatment. NPS: nasal polyp score (range 0–8, both nostrils simultaneously); SNOT-22: sinonasal outcome test-22 items (0–110); VAS: visual analog scale (0–10).

The cohort showed a statistically significant reduction in nasal polyp size (mean change: −2.56 [−3.24, −1.88], *p* < 0.0001) after six months of mepolizumab treatment (Table 2, Figure 1). Significant and large-size effect improvements were symmetrically observed in both nostrils (Figure S2). The proportion of patients achieving an improvement score of ≥1 or ≥2 in total NPS was 85% and 78% respectively, with 43% showing a remarkable improvement of ≥4 points after six months of therapy (Figure S3). The proportion of patients that improved ≥1 point in the nasal polyp score in the left nostril and ≥1 point in the right nostril simultaneously was 66%. A reduction of ≥1 point in the total NPS is considered the minimal clinically important difference (MCID) [3,6] and was achieved by 85% of the patients in our cohort.

### 3.3. SNOT-22, Nasal Congestion VAS, and Smell Impairment VAS

Mean SNOT-22 score improved 25.29 points after six months of therapy (*p* < 0.0001) (Table 2, Figure 1). Around 76% of the patients achieved an improvement above the defined minimal clinically important difference (MCID) threshold for the questionnaire (MCID > 8.9). Assessment of the SNOT-22 by domains also led to significant improvements for each of the domains (Table S1, Figure S4). Interestingly, significant improvements were observed for each of the items displayed on the SNOT-22, with only one item of the twenty-two assessed (4%) showing a small effect size (Table S2).

**Figure 2:**
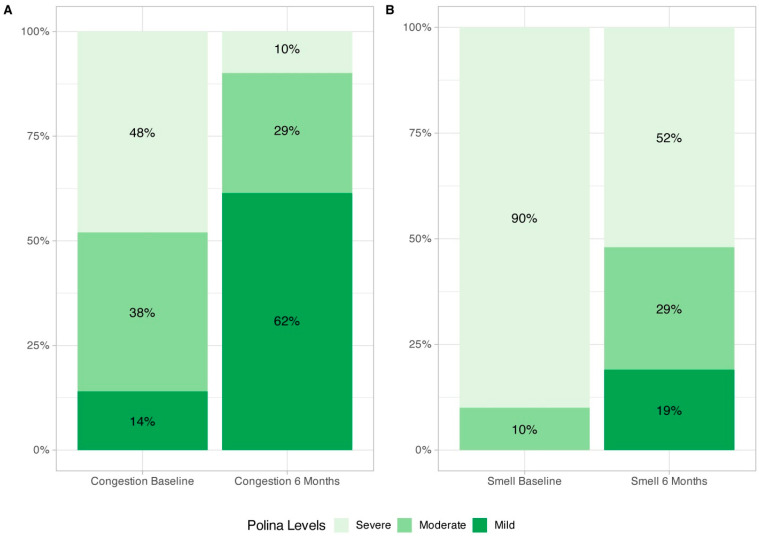
Proportion of patients with mild, moderate, and severe nasal congestion VAS (**A**) and smell impairment VAS (**B**) at baseline and after six months of mepolizumab treatment. Staging of smell impairment and nasal congestion impairment severity according to VAS: mild (VAS ≤ 3), moderate (3 < VAS ≤ 7), and severe (VAS > 7). VAS: visual analog scale (0 to 10).

Nasal congestion (−3.57 [−4.50, −2.65], *p* < 0.0001) and smell impairment (−4.0 [−5.50, −3.0], *p* < 0.0001) VAS showed a significant improvement after six months of treatment (Table 2, Figure 1). The proportion of patients reporting severe nasal congestion decreased from 48% at baseline to 10% after 6 months of treatment. Similarly, the percentage of patients with severe olfactory dysfunction decreased from 90% at baseline to 52% at 6 months. These findings are illustrated in Figure 2.

### 3.4. ESS Events, SCS Intake, and Inflammatory Biomarkers

Only one patient (2%) required ESS, and 85% subjects did not require SCS prescription for the management of CRSwNP after mepolizumab initiation. Blood and tissue eosinophil median counts showed a significant reduction of 86% and 26% respectively versus baseline (Table 2).

### 3.5. Asthma Control

Among those patients with a concomitant diagnosis of asthma the median ACT score increased by 8 points from baseline to 6 months after treatment, achieving a median value of 24 points (Table 2). An improvement of at least 3 points, the validated MCID for this test, was achieved by 65% of the subjects with CRSwNP and asthma. At baseline, only 35% of the subjects were classified as controlled (ACT ≥ 20); however, after six months of mepolizumab treatment, this number increased to 84%. No statistically significant differences were observed in ACT improvements between severe versus non-severe asthma patients (Table S3).

### 3.6. Safety and Treatment Discontinuation Events

Three patients experienced adverse effects that were resolved spontaneously: one patient reported arthralgia, one patient reported myalgia, and another patient reported mild rhinitis symptoms. Two patients abandoned treatment for reasons different to lack of efficacy or safety: one patient disliked using an injectable chronic treatment and opted out after four months, and another patient suspended treatment after five months as per suggestion by another healthcare professional.

## 4. Discussion

The findings from this study provide valuable insights into the real-world effectiveness of mepolizumab in a diverse population of patients with CRSwNP. The results demonstrate significant improvements in patient-reported outcomes, nasal polyp score, and asthma control after six months of treatment, as well as in the reduction of blood and tissue eosinophil counts, highlighting the potential of mepolizumab as a therapeutic option for this patient population and expanding on the existent data from randomized controlled studies and previous findings in clinical settings. These results are in line with those reported in previous clinical and real-world studies assessing the role of mepolizumab in patients with type 2 inflammatory CRSwNP [7,8,9,10,11].

An important strength of this study is the heterogeneity of the patient population included. Beyond the presence of patients with both severe and non-severe asthma, our cohort comprised individuals with a wide spectrum of disease characteristics: patients with and without N-ERD, varying degrees of systemic and tissue eosinophilia, and different histories regarding systemic corticosteroid exposure (including both frequent and minimal prior use). Surgical history was also diverse, ranging from patients who had never undergone endoscopic sinus surgery (ESS) to those with multiple prior interventions. Additionally, the age distribution, time from diagnosis to biologic initiation, and baseline symptom burden (as reflected by SNOT-22) showed substantial variation across the sample.

This clinical diversity reflects real-world practice more accurately than the homogeneous populations often selected for randomized controlled trials, and it enhances the external validity of our findings regarding the impact of mepolizumab on CRSwNP management.

To the best of our knowledge this study evaluated, for the first time, every single item on the SNOT-22 as well as the improvement in the SNOT-22 domain scores in clinical settings, offering precise evidence regarding mepolizumab symptom management. This comprehensive analysis of the SNOT-22 items and domains provides nuanced information on the impact of mepolizumab on specific symptom dimensions, thus contributing uniquely to the current body of literature. Previous studies have focused predominantly on total scores or general QoL improvement; in contrast, we aimed to dissect the response across all domains (rhinologic, sleep, psychological, etc.) and even individual items, highlighting the real-world benefit as perceived by patients.

A large proportion of patients achieved clinically relevant QoL improvements, and the most burdensome symptoms to patients were among the items that showed the largest improvements after six months of mepolizumab treatment. Improvement of smell impairment and nasal congestion VAS were also consistent with data observed in SNOT-22 scores. It is worth highlighting the reduction in symptom burden according to VAS cut-offs, with 48% and 90% of patients classified as non-severe for smell impairment and congestion symptoms, respectively. These combined data suggest that mepolizumab improves patient perception about the burden of the disease and quality of life status. Our results of improvement in total SNOT-22 and smell and congestion VAS are in line with previous evidence in clinical settings by other research groups, such as Orlando and Cavaliere [10,11]. However, Dominguez et al. reported a numerically larger mean improvement of 63 points in SNOT-22 [9]. In this study, all patients had severe asthma, whereas in our study the proportion of patients with non-severe asthma was 56%. In some studies, such as that of Farhood et al. [19], asthma has been reported as a comorbidity that increases SNOT-22 scores, supporting the single airway theory described by Krouse et al. [20]. For this reason, we believe that the coexistence of severe asthma with CRSwNP may have influenced the poorer baseline SNOT-22 score in the population of the study by Dominguez et al. and, at the same time, contributed to a numerically larger improvement following treatment compared to our findings.

The substantial reduction in total NPS after six months of therapy is particularly noteworthy. Data showed that more than 80% of patients experienced at least a 1-point improvement in NPS, considered in the literature as the MCID for this endpoint. As many as 50% of patients achieved even greater reductions beyond 3 points. This study reported also the evaluation of the simultaneous improvement in the left and in the right nostril. In fact, most of the patients that achieved a NPS improvement of at least two points was because of a concomitant, simultaneous reduction of one point in the left nostril and one point in the right nostril. Such simultaneous improvements in both nostrils are likely contributing to the observed large improvements in nasal congestion. Orlando et al. showed that around 70% of the patients treated with mepolizumab achieved a NPS improvement of at least two points after one year, whereas the studies from Dominguez and Cavaliere displayed similar NPS reductions to what we have observed, highlighting the consistent benefits of mepolizumab in objectively reducing polyp size in clinical settings [9,10,11].

Improvements in ACT scores highlight the dual benefit of an anti-IL-5 biologic therapy in patients with CRSwNP and comorbid type 2 asthma. Phase III SYNAPSE post-hoc studies evaluating the subgroup of patients with severe CRSwNP and asthma demonstrated a concomitant benefit of this therapy in lower airways [21]. Those results are corroborated in real-world settings, although most of the reports included a large proportion of patients with comorbid severe asthma [9,10,11]. Our study evaluated a more diverse patient population beyond those with comorbid severe asthma. After six months of mepolizumab initiation, the CRSwNP plus asthma study population group showed a statistically significant and clinically relevant improvement in asthma control, whereas no statistically significant differences were observed in asthma control improvements between both sub-groups of severe and non-severe asthmatics (Table S3). The systemic and local reduction blood and tissue eosinophil counts are likely playing a key role for the observed benefits in this patient population. Actually, the reported findings may be explained due to the paramount importance of IL-5 in type 2 inflammation, a pleiotropic cytokine that not only plays a role in eosinophil activation and recruitment, but also in increasing mucus thickness, promoting immune disbalance, accelerating nasal polyp recurrence, and contributing to tissue remodeling [22]. Hence, anti-IL-5 gents are suggested to be a suitable treatment to reduce the clinical manifestations derived from type 2 inflammation in both upper and lower airways [23].

This piece of research was designed as an observational multicenter retrospective study, yet it has several limitations such as missing data bias due to the nature of the recollection of data and lack of a control arm. Also, the relatively short follow-up period of six months may not capture long-term outcomes and potential late-onset adverse events. On the contrary, its multicentric design reinforces the external validity of the data, as bias from a monocentric observational design is likely avoided. Our population, although relatively limited in size, is comparable to other real-world studies in this field. Importantly, the diversity of our cohort lies not only in the multicentric design, but also in the inclusion of patients with both severe and non-severe asthma, varying degrees of systemic corticosteroid exposure, and differing surgical histories—all within the context of features suggestive of type 2 inflammatory endotype.

Certain specific characteristics of our study population warrant consideration. Notably, the proportion of individuals diagnosed with nonsteroidal anti-inflammatory drug-exacerbated respiratory disease (N-ERD) was relatively high. This can be attributed to the selection criteria for biologic therapy in our healthcare setting, which primarily targets patients with more severe disease phenotypes, specifically those presenting with CRSwNP, asthma, and nonsteroidal anti-inflammatory drugs intolerance.

One limitation of our study is the absence of systematic radiological assessment using Lund–Mackay scores. In line with real-world clinical practice, CT imaging was only performed when clinically justified (e.g., prior to surgical intervention) and not repeated routinely after treatment due to concerns about unnecessary radiation exposure. As a result, radiologic comparisons were not feasible for the entire cohort.

A distinctive aspect of our national healthcare system is that it remains the only one within our regional context to mandate a minimum of two prior surgical interventions for nasal polyposis as a prerequisite for public reimbursement of biologic therapy. Patients who did not undergo surgery prior to the initiation of mepolizumab treatment were those for whom surgical intervention was contraindicated due to specific clinical considerations. In all such instances, the decision to proceed with biologic therapy without prior surgery was endorsed by the corresponding institutional multidisciplinary committees responsible for evaluating biologic therapies in the context of severe respiratory disease.

## 5. Conclusions

This study provides real-world evidence supporting the effectiveness of mepolizumab in managing CRSwNP in patients with features suggestive of type 2 inflammation. The observed improvements in patient-reported outcomes, nasal polyp burden, and asthma control suggest that mepolizumab may be a valuable therapeutic option for this patient population. While our findings are limited by the observational design and sample size, they contribute to the growing body of evidence supporting biologic therapy in type 2 CRSwNP. Further research in larger and more diverse cohorts is warranted to confirm these results and explore long-term outcomes.

## Figures and Tables

**Table 1:** Patients baseline features.

Variables		N = 47
Age (years)	Med [IQR]	56.0 [45.5, 63.0]
	Mean (std)	54.3 (11.7)
Sex (n, %)	Men	24 (51.06%)
	Women	23 (48.94%)
Atopy	No	25 (53.19%)
	Yes	22 (46.81%)
Total IgE (UI/mL)	Med [IQR]	113.0 [52.0, 270.5]
	Mean (std)	248.1 (352.9)
Age at CRSwNP diagnosis (years)	Med [IQR]	40.0 [29.0, 45.0]
	Mean (std)	38.4 (11.6)
Number of ESS	Med [IQR]	2.0 [1.0, 3.0]
	Mean (std)	2.4 (2.4)
Proportion of patients with 0, 1, >1 ESS: n (%)	0 surgeries	8 (17.02%)
	1 surgery	7 (14.89%)
	>1 surgery	32 (68.09%)
Time from CRSwNP diagnosis to mepolizumab initiation (months)	Med [IQR]	159.0 [72.0, 240.0]
	Mean (std)	176.3 (128.6)
Time from last surgery to mepolizumab initiation (months)	Med [IQR]	48.0 [12.0, 89.2]
	Mean (std)	59.8 (61.9)
N-ERD, n (%)	No	17 (36.17%)
	Yes	30 (63.83%)
Asthma, n (%)	No	4 (8.51%)
	Yes	43 (91.49%)
Asthma severity, n (%)	Mild	9 (20.93%)
	Moderate	15 (34.88%)
	Severe	19 (44.19%)
Intranasal corticosteroid treatment, n (%)	Yes	47 (100.00%)
	No	0 (0%)
Saline rinses, n (%)	No	14 (29.79%)
	Yes	33 (70.21%)
Systemic corticosteroid courses in the previous 12 months	Med [IQR]	2.0 [1.0, 3.0]
	Mean (std)	1.9 (1.7)
Proportion of patients with 0, 1 >1 courses, n (%)	0 courses	9 (19.15%)
	1 course	12 (25.53%)
	>1 course	26 (55.32%)

Legend: Med [IQR]: Median and interquartile range; Mean (std): mean and standard deviation. IgE: immunoglobulin E; CRSwNP: chronic rhinosinusitis with nasal polyps; ESS: endoscopic sinus surgery; N-ERD: non-steroidal anti-inflammatory drug-exacerbated respiratory disease.

**Table 2:** Assessed endpoints at baseline and after six months of mepolizumab treatment.

		Baseline	6 Months	*p* Value	Median or Mean Difference (IC95%) ^3^	Effect Size ^4^
Total NPS	Med [IQR]	6.0 [4.0, 6.0]	2.0 [2.0, 4.0]	<0.0001 ^2^	−2.56 [−3.24, −1.88]	−1.42 (large) [−1.94, −0.89]
	Mean (std)	5.1 (1.7)	2.7 (1.9)			
SNOT-22	Med [IQR]	56.0 [41.0, 74.0]	23.0 [14.5, 47.5]	<0.0001 ^2^	−25.29 [−32.15, −18.42]	1.06 (large) [−1.42, −0.71]
	Mean (std)	55.1 (25.1)	30.0 (21.1)			
Nasal congestion VAS	Med [IQR]	7.0 [5.0, 9.0]	2.0 [1.0, 4.8]	<0.0001 ^2^	−3.57 [−4.50, −2.65]	−1.34 (large) [−1.81, −0.87]
	Mean (std)	6.6 (2.5)	3.0 (2.7)			
Smell impairment VAS	Med [IQR]	10.0 [9.0, 10.0]	8 [4.0, 10.0]	<0.0001 ^1^	−4.0 [−5.50, −3.0]	−0.96 (large) [−1.39, −0.54]
	Mean (std)	9.2 (1.5)	6.5 (3.4)			
Blood eosinophil count *	Med [IQR]	670.0 [500.0, 937.5]	95.0 [60.0, 180.0]	<0.0001 ^1^	−625.45 [−770.0, −495.0]	−1.41 (large) [−1.98, −0.84]
	Mean (std)	829.8 (619.4)	155.5 (196.7)			
Tissue eosinophil count **	Med [IQR]	51.0 [36.2, 187.5]	37.5 [6.2, 70.0]	<0.0001 ^1^	−52.5 [−119.9, −25.0]	−0.69 (medium) [−1.17, −0.20]
	Mean (std)	125.8 (131.3)	51.1 (69.4)			
ACT	Med [IQR]	15.0 [11.0, 21.0]	24.0 [21.0, 25.0]	<0.001 ^1^	8.0 [5.50, 11.99]	1.34 (large) [0.71, 1.97]
	Mean (std)	15.2 (6.9)	22.2 (3.7)			

Legend: ^1^ Wilcoxon signed rank test with continuity correction. ^2^ Paired samples *t*-test. ^3^ Hodges–Lehmann Estimator (95% CI). ^4^ Cohen’s D with Hedge’s Correction. Med [IQR]: median and interquartile range. Mean (std): mean and standard deviation. NPS: nasal polyp score (range 0–8, both nostrils simultaneously); SNOT-22: sinonasal outcome test-22 items (0–110); VAS: visual analog scale (0–10); * Blood eosinophil count (cells per microliter of blood); ** Tissue eosinophil count (number of eosinophils per 10 high-power fields); ACT: asthma control test (0–24).

## Data Availability

The data presented in this study are available on request from the corresponding authors.

## Supplementary Materials

The following supporting information can be downloaded at: https://www.mdpi.com/article/10.3390/jcm14155248/s1, Figure S1: The most burdensome symptoms ranked by order, based on baseline SNOT-22 frequency of selection. SNOT-22: Sinonasal Outcome Test-22 items; Item 21 (11.9%): Loss of taste/smell; Item 1 (11.4%): Need to blow nose; Item 22 (10.8%): Nasal congestion; Item 3 (8%): Runny nose; Item 6 (8%): Thick nasal discharge. Figure S2: Left (L) and right (R) nostril NPS at baseline and after six months of mepolizumab treatment. NPS: nasal polyp score (range 0–4, each nostril). Figure S3: Proportion of patients achieving a total NPS difference of ≥1, ≥2, ≥3, or ≥4 after six months of mepolizumab treatment. NPS: nasal polyp score (range 0–8, both nostrils simultaneously). Figure S4: SNOT-22 domain improvement at baseline and after six months of mepolizumab treatment: (A) Rhinologic domain, (B) Ear/face domain, (C) Sleep domain, (D) Extra-rhinologic domain, (E) Psychologic domain, (F) Total SNOT-22 Score. SNOT-22: sinonasal outcome test-22 items (0–110). Table S1: Assessment of SNOT-22 domain improvements at baseline and after six months of mepolizumab treatment. ^1^Paired samples *t*-test. ^2^Hodges–Lehmann estimator (95% CI). ^3^Cohen’s D with Hedges correction. SNOT-22: sinonasal outcome test-22 items (0–110). ^1^Wilcoxon signed rank test with continuity correction. ^2^Paired samples *t*-test. ^3^Hodges–Lehmann estimator (95% CI). ^4^Cohen’s D with Hedges correction. SNOT-22: sinonasal outcome test-22 items (0–110). Table S2: SNOT-22 items improvements after six months of mepolizumab treatment. Table S3: Assessment of ACT differences in the two subgroups of CRSwNP asthmatic patients, with and without severe asthma, after six months of mepolizumab treatment. ^1^Wilcoxon signed rank test with continuity correction. ACT: asthma control test (0–24).

## Abbreviations

The following abbreviations are used in this manuscript:

CRSwNP	chronic rhinosinusitis with nasal polyps
QoL	quality of life
SCS	systemic corticosteroids
ESS	endoscopic sinus surgery
mAb	monoclonal antibodies
NPS	nasal polyp score
SNOT-22	Sinonasal Outcome Test-22 items
VAS	visual analog scale
ACT	Asthma Control Test
MCID	minimal clinically important difference
N-ERD	non-steroidal anti-inflammatory drug-exacerbated respiratory disease
