# Supplementary material for: Transforming Patient Experience: Real-World Impact of Mepolizumab on Symptom Burden in Chronic Rhinosinusitis with Nasal Polyps—A Multicenter Perspective
Source: J Clin Med. 2025 Jul 24;14(15):5248. doi: 10.3390/jcm14155248 (PMC12347621; doi:10.3390/jcm14155248)
Supplement: Supplementary file 1 [file jcm-14-05248-s001.zip › jcm-3681936-supplementary.pdf]

Supplementary information for:

Transforming patient experience: Real-world impact of mepolizumab on symptom burden in chronic rhinosinusitis with nasal polyps - A multicenter perspective.

Figure S1: The most burdensome symptoms ranked by order, based on baseline SNOT-22 frequency of selection.

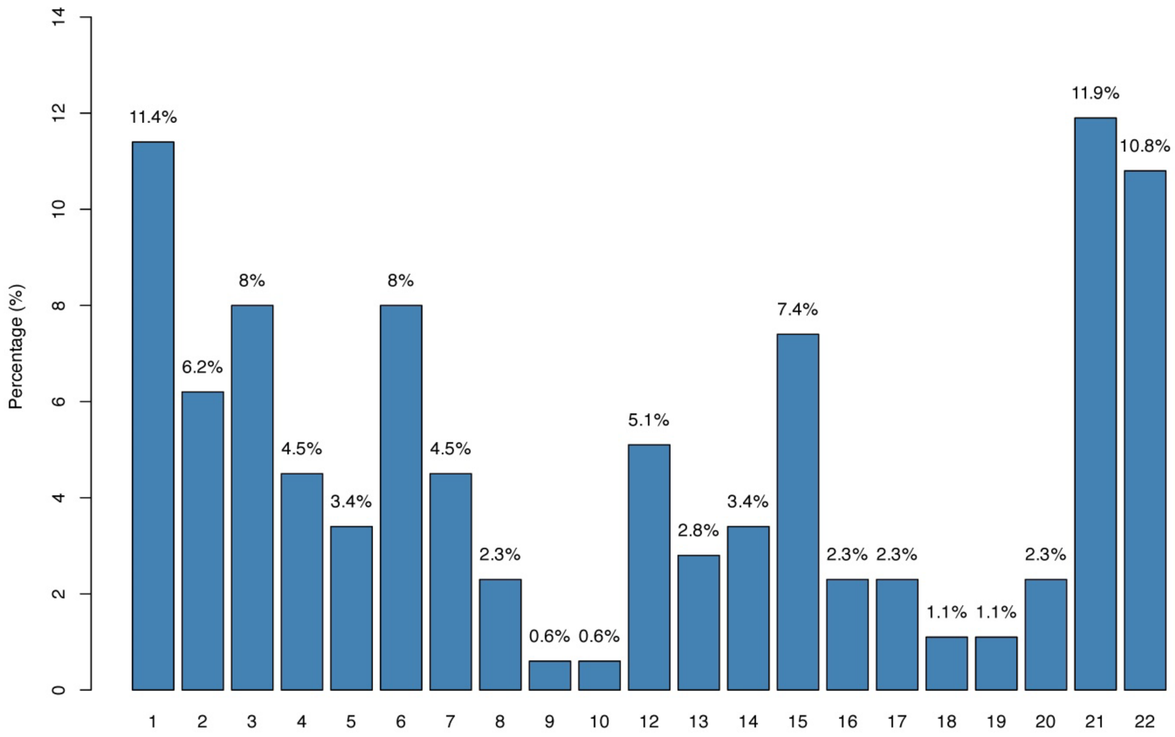

SNOT-22: Sinonasal Outcome Test-22 items; Item 21 (11,9%): Loss of taste/smell; Item 1 (11,4%): Need to blow nose; Item 22 (10,8%): Nasal congestion; Item 3 (8%): Runny nose; Item 6 (8%): Thick nasal discharge.

Figure S2: Left (L) and Right (R) nostril NPS at baseline and after six months of mepolizumab treatment.

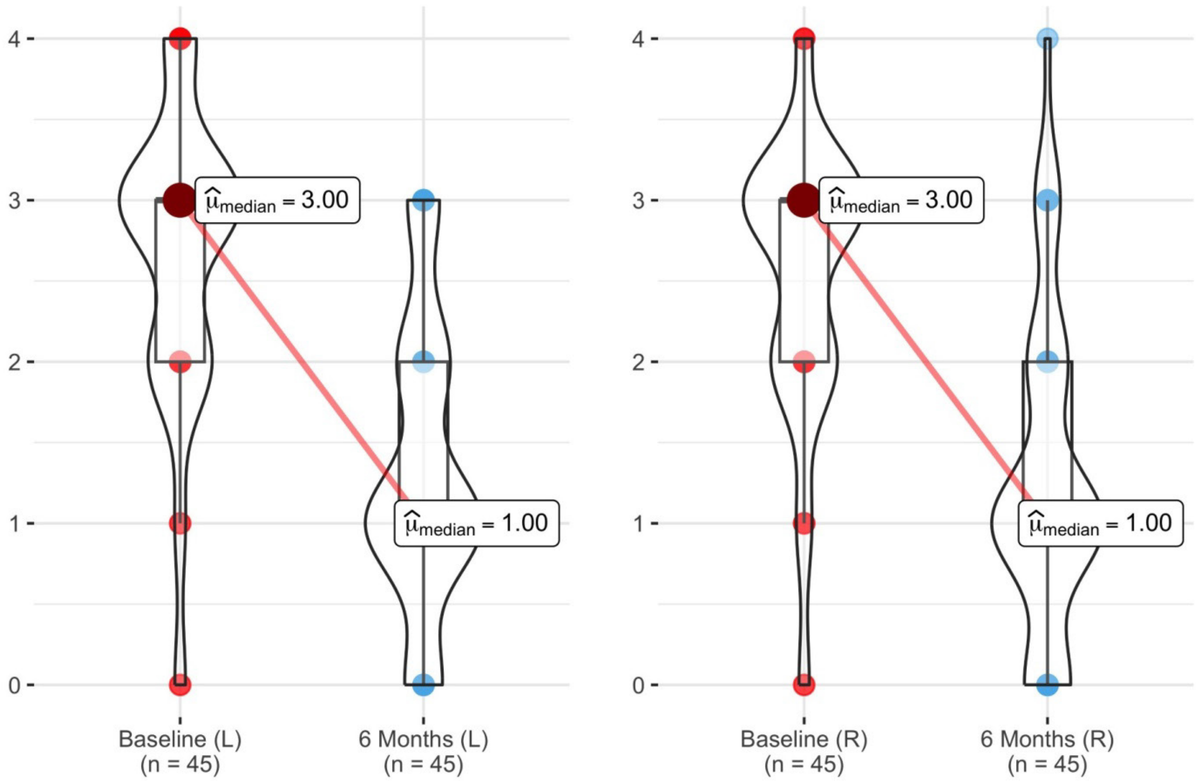

NPS: nasal polyp score (range 0-4, each nostril)

Figure S3: Proportion of patients achieving a total NPS difference of ≥1, ≥2, ≥3, ≥4 after six months of mepolizumab treatment.

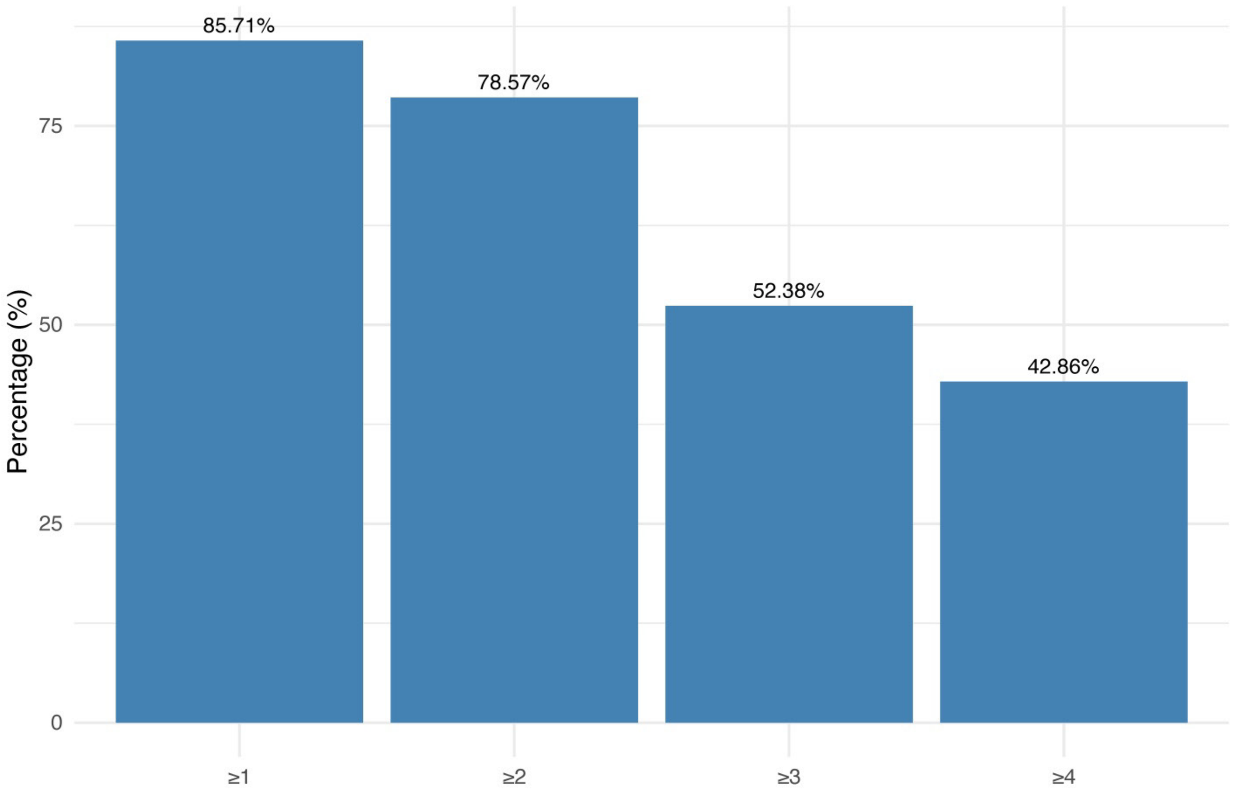

NPS: nasal polyp score (range 0-8, both nostrils simultaneously).

Figure S4: SNOT-22 domain improvement at baseline and after six months of mepolizumab treatment: A) Rhinologic domain, B) Ear/face domain, C) Sleep domain, D) Extra-rhinologic domain, E) Psychologic domain, F) Total SNOT-22 Score.

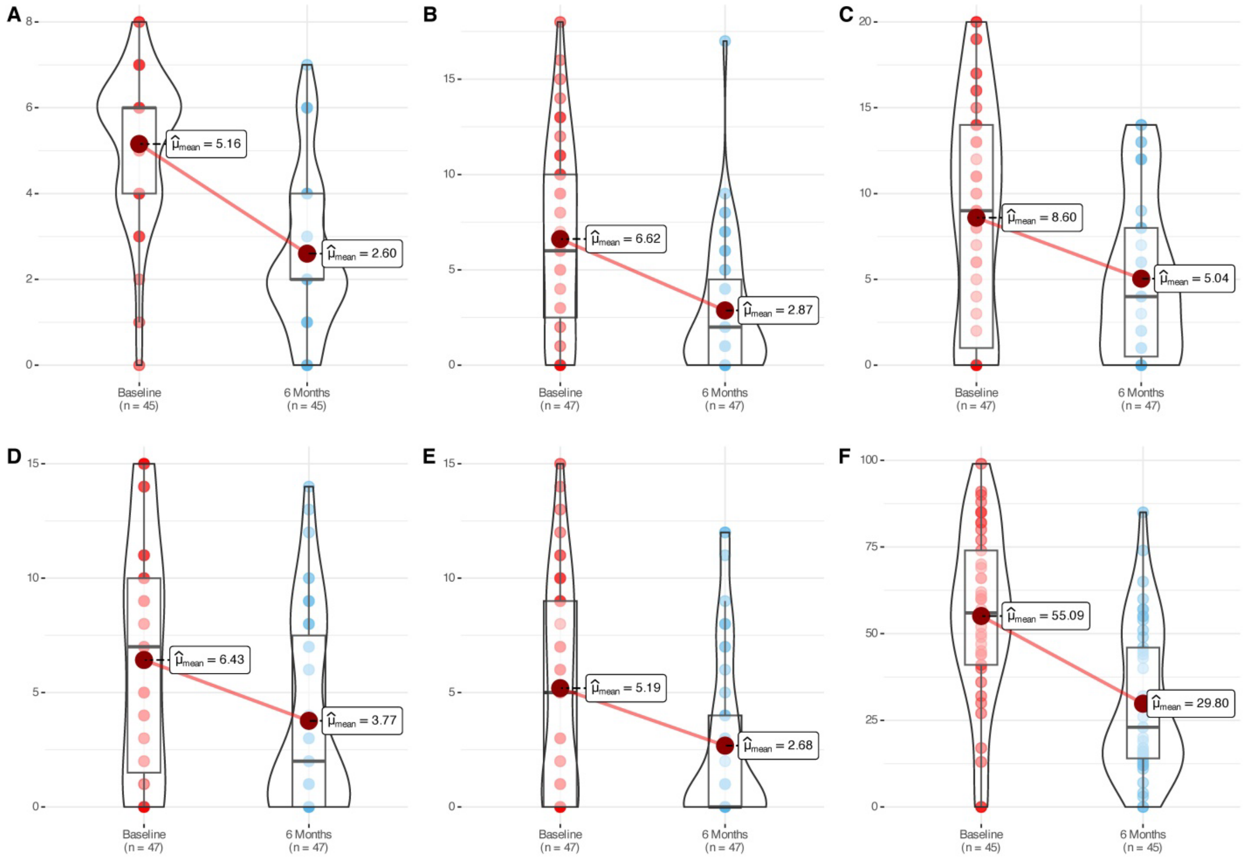

SNOT-22: sinonasal outcome test-22 items (0-110)

Table S1: Assessment of SNOT-22 domain improvements at baseline and after six months of mepolizumab treatment.

Domain	Baseline		6 Months		P-value[1]	Mean difference[2]	Effect Size[3]
Rhinologic domain	Med [IQR]	26.0 [13.5;30.0]	Med [IQR]	11.0 [6.0;18.0]	<0.0001	-8.74 [-11.82, -5.66]	-0.76 (medium) [-1.06, -0.46]
	Mean (std)	21.3 (12.4)	Mean (std)	12.6 (9.5)			
Ear/face domain	Med [IQR]	6.0 [2.5;10.0]	Med [IQR]	2.0 [0;4.5]	<0.0001	-3.74 [-5.05, -2.43]	-0.82 (large) [-1.15, -0.49]
	Mean (std)	6.6 (5.1)	Mean (std)	2.9 (3.4)			
Sleep domain	Med [IQR]	9.0 [1.0;14.0]	Med [IQR]	4.0 [0.5;8.0]	<0.0001	-3.55 [-5.21, -1.89]	-0.59 (medium) [-0.89, -0.29]
	Mean (std)	8.6 (6.6)	Mean (std)	5.0 (4.6)			
Extra-rhinologic domain	Med [IQR]	7.0 [1.5;10.0]	Med [IQR]	2.0 [0;7.5]	<0.0001	-2.66 [-3.93, -1.38]	-0.55 (medium) [-0.83, -0.27]
	Mean (std)	6.4 (5.1)	Mean (std)	3.8 (4.3)			
Psychologic domain	Med [IQR]	5.0 [0;9.0]	Med [IQR]	0 [0;4.0]	<0.0001	-2.51 [-3.67, -1.35]	-0.58 (medium) [-0.87, -0.29]
	Mean (std)	5.2 (4.6)	Mean (std)	2.7 (3.6)			

[1]Paired samples T-test. [2]Hodges–Lehmann Estimator (95% CI). [3]Cohen's D with Hedges Correction. SNOT-22: sinonasal outcome test-22 items (0-110).

Table S2: SNOT-22 items improvements after six months of mepolizumab treatment.

SNOT-22 item		**Baseline**	**6 Months**	**P-value**	**Median or mean difference IC95% [3]**	**Effect Size[4]**
1.Need to blow nose	Med [IQR]	4.0 [3.0;4.0]	2.0 [1.0;3.0]	<0.0001[1]	-2.0 [-2.5, -1.50]	-1.07 (large) [-1.53, -0.59]
	Mean (std)	3.5 (1.2)	2.0 (1.4)			
2.Sneezing	Med [IQR]	3.0 [2.0;4.0]	1.5 [1.0;3.0]	<0.0001[1]	-1.50 [-2.0, -1.0]	-0.80(medium) [-1.12, -0.47]
	Mean (std)	3.0 (1.5)	1.8 (1.5)			
3.Runny nose	Med [IQR]	3.0 [2.0;4.0]	1.0 [1.0;3.0]	0.00029[1]	-1.50 [-2.0, -0.99]	-0.76(medium) [-1.16, -0.36]
	Mean (std)	3.0 (1.6)	1.8 (1.5)			
4. Cough	Med [IQR]	3.0 [2.0;4.0]	1.0 [1.0;2.0]	<0.0001[1]	-1.50 [-1.99 -1.0]	-0.94 (large) [-1.37, -0.51]
	Mean (std)	2.9 (1.4)	1.5 (1.2)			
5. Post nasal discharge	Med [IQR]	3.0 [2.0;4.0]	1.0 [0;2.0]	<0.0001[1]	-1.99 [-2.50, -1.0]	-0.97 (large) [-1.4, -0.52]
	Mean (std)	2.8 (1.5)	1.3 (1.4)			
6. Thick nasal discharge	Med [IQR]	4.0 [3.0;5.0]	2.0 [1.0;3.0]	<0.0001[1]	-2.0 [-2.50, -1.50]	1.33 (large) [-1.87 -0.79]
	Mean (std)	3.7 (1.2)	1.8 (1.3)			
7. Ear fullness	Med [IQR]	3.0 [2.0;4.0]	1.0 [0;2.0]	<0.0001[1]	-2.0 [-2.50, -1.50]	-1.17 (large) [-1.69, -0.64]
	Mean (std)	2.9 (1.5)	1.1 (1.2)			
8. Dizziness	Med [IQR]	2.0 [0.2;3.0]	0 [0;2.0]	0.00156[1]	-1.0 [-1.99, -0.99]	-0.53(medium) [-0.87 -0.20]
	Mean (std)	1.9 (1.6)	1.0 (1.5)			
9. Ear pain/pressure	Med [IQR]	1.0 [0;2.0]	0 [0;1.0]	0.00088[1]	-1.50 [-2.0, -1.0]	-0.79(medium) [-1.24, -0.34]
	Mean (std)	1.4 (1.5)	0.4 (0.9)			
10. Facial pain/pressure	Med [IQR]	2.0 [0;3.0]	0 [0;1.0]	<0.0001[1]	-1.99 [-2.49, -1.49]	-0.84 (large) [-1.21, -0.48]
	Mean (std)	1.9 (1.7)	0.6 (1.0)			
11. Difficulty falling asleep	Med [IQR]	2.0 [1.0;3.0]	0.5 [0;2.0]	0.00013[1]	-1.50 [-2.0, -1.0]	-0.79(medium) [-1.17, -0.42]
	Mean (std)	2.2 (1.6)	1.0 (1.2)			
12. Waking up at night	Med [IQR]	3.0 [2.0;4.0]	1.5 [0.2;4.0]	0.01106[1]	-1.50 [-2.49, -0.49]	-0.42 (small) [-0.75, -0.09]
	Mean (std)	2.9 (1.7)	2.1 (1.9)			
13. Lack of a good night's sleep	Med [IQR]	3.0 [1.0;4.0]	1.0 [0;2.0]	<0.0001[1]	-2.0 [-2.50, -1.50]	-1.09 (large) [-1.53, -0.66]
	Mean (std)	2.7 (1.7)	1.1 (1.2)			
14. Waking up tired	Med [IQR]	3.0 [2.0;4.0]	1.0 [0;3.0]	0.00022[1]	-1.99 [-2.49, -1.0]	-0.82 (large) [-1.25, -0.39]
	Mean (std)	2.9 (1.6)	1.5 (1.5)			
15. Fatigue during the day	Med [IQR]	3.5 [1.2;4.8]	1.0 [0;3.0]	<0.0001[1]	-1.50 [-2.0, -1.0]	-0.81 (large) [-1.13, -0.48]
	Mean	3.1 (1.7)	1.5 (1.6)			

	(std)					
16. Reduced productivity	Med [IQR]	2.5 [1.0;3.8]	1.0 [0;2.8]	0.00026[1]	-1.49 [-1.99, -0.99]	-0.62(medium) [-0.94, -0.29]
	Mean (std)	2.5 (1.6)	1.4 (1.5)			
17. Reduced concentration	Med [IQR]	3.0 [1.0;3.0]	1.0 [0;2.8]	0.00055[1]	-1.50 [-2.0, -0.99]	-0.6401674 (medium) [-0.98, -0.29]
	Mean (std)	2.4 (1.6)	1.3 (1.4)			
18. Frustrated / restless / irritable	Med [IQR]	3.0 [1.2;4.0]	0 [0;2.8]	<0.0001[1]	-1.99 [-2.50, -1.0]	-0.80 (large) [-1.14 -0.47]
	Mean (std)	2.6 (1.6)	1.2 (1.6)			
19. Sad	Med [IQR]	3.0 [1.0;4.0]	0 [0;2.0]	<0.0001[1]	-2.0 [-2.99, -1.49]	-0.83 (large) [-1.19, -0.45]
	Mean (std)	2.4 (1.7)	1.0 (1.4)			
20. Embarrassed	Med [IQR]	1.0 [0;2.0]	0 [0;1.0]	0.00061[1]	-1.50 [-2.0, -1.0]	-0.52(medium) [-0.80, -0.23]
	Mean (std)	1.5 (1.6)	0.7 (1.1)			
21. Sense of taste / smell	Med [IQR]	5.0 [3.0;5.0]	2.0 [0;3.0]	<0.0001[1]	-2.50 [-3.0, -1.99]	-1.03 (large) [-1.48, -0.59]
	Mean (std)	3.8 (1.7)	2.0 (1.8)			
22. Blockage / congestion of nose	Med [IQR]	4.0 [3.0;5.0]	1.5 [0;3.0]	<0.0001[1]	-2.50 [-2.99, -1.99]	-1.08 (large) [-1.50, -0.67]
	Mean (std)	3.7 (1.5)	1.8 (1.6)			
TOTAL	Med [IQR]	56.0 [41.0;74.0]	23.0 [14.5;47.5]	<0.0001[2]	-25.29 [-32.15, -18.42]	1.06 (large) [-1.42, -0.71]
	Mean (std)	55.1 (25.1)	30.0 (21.1)			

[1]Wilcoxon signed rank test with continuity correction. [2] Paired samples T-test. [3]Hodges–Lehmann Estimator (95% CI). [4]Cohen's D with Hedges Correction. SNOT-22: sinonasal outcome test-22 items (0-110).

Table S3: Assessment of ACT differences in the two subgroups of CRSwNP asthmatic patients, with and without severe asthma, after six months of mepolizumab treatment.

		Non-Severe	Severe	P-value	Median or mean difference (IC95%) [3]	Effect Size[4]
**ACT improvement**	Med [IQR]	7.0 [2.0;15.0]	7.0 [1.0;9.0]	0.4972[1]	-1 [-7, 4]	0.33 (small) [-0.36, 1.02]
	Mean (std)	8.7 (8.6)	6.1 (5.8)			

[1]Wilcoxon signed rank test with continuity correction. ACT: asthma control test (0-24)
